# What Happens When the Honeymoon is Over? Sustaining Community Partner Relationships After Recruitment

**DOI:** 10.1002/alz.087569

**Published:** 2025-01-09

**Authors:** Melita H Terry, Lilcelia A. Williams, C. Elizabeth Shaaban, Jennifer H Lingler

**Affiliations:** ^1^ University of Pittsburgh Alzheimer’s Disease Research Center (ADRC), Pittsburgh, PA USA; ^2^ Department of Occupational Therapy, University of Pittsburgh, School of Health and Rehabilitation Sciences, Bridgeside Point I 100 Technology Drive, PA USA; ^3^ University of Pittsburgh, Pittsburgh, PA USA

## Abstract

**Background:**

Increasing calls for diversity in Alzheimer’s disease and related dementias (ADRDesearch have fueled significant investments in recruitment personnel and activities. However, the essential work of identifying and authentically engaging communities of color in relationship‐building begins well before, and continues long after, study recruitment activities take place. The purpose of this presentation is twofold: 1) to differentiate the work of trust‐building and relationship cultivation from that of research study recruitment, and 2) to describe the necessary steps to ensure that relationship cultivation is ongoing and supported beyond the timeline of a singular research study.

**Method:**

This presentations draws on our team’s experience developing, implementing, and evaluating community engagement programming at a federally funded Alzheimer’s Disease Research Center (ADRC). We outline key features of relationship cultivation and contrast these efforts from the narrower, yet related, activity of study recruitment. We will offer real‐life exemplars to delineate the steps necessary for effective relationship cultivation, particularly in a post‐pandemic era. The importance of intentional actions to sustain partnerships, programming and participant relationships beyond the research recruitment phase will be demonstrated. The relevance of this work for individuals along the spectrum of participant readiness to enroll in ADRD research or consent to brain donation will be explained.

**Result:**

Relationship cultivation efforts at our Center have led to: a) sustained community partnerships, including a multi‐decade presence in a predominantly Black and African‐American neighborhood despite changes in local governance, businesses, and service organizations; b) sustained programming, including a lecture series honoring a prominent local historical figure; and c) sustained longitudinal research participation among individuals identifying as Black or African American, as exemplified by a case study of a participant who was active in our cohort for 28 years and ultimately provided brain donation.

**Conclusion:**

The value of relationship building as the primary step for powerful community engagement and participant partnership should not be underestimated by researchers. This work should be considered a critical aspect of research infrastructure. The foundation laid by building and sustaining the meaningful relationships is essential to bridging trust between community members and ADRD researchers and continues long beyond the recruitment honeymoon.

